# Peak Width of Skeletonized Water Diffusion MRI in the Neonatal Brain

**DOI:** 10.3389/fneur.2020.00235

**Published:** 2020-04-03

**Authors:** Manuel Blesa, Paola Galdi, Gemma Sullivan, Emily N. Wheater, David Q. Stoye, Gillian J. Lamb, Alan J. Quigley, Michael J. Thrippleton, Mark E. Bastin, James P. Boardman

**Affiliations:** ^1^MRC Centre for Reproductive Health, University of Edinburgh, Edinburgh, United Kingdom; ^2^Department of Radiology, Royal Hospital for Sick Children, Edinburgh, United Kingdom; ^3^Centre for Clinical Brain Sciences, University of Edinburgh, Edinburgh, United Kingdom; ^4^Edinburgh Imaging, University of Edinburgh, Edinburgh, United Kingdom

**Keywords:** diffusion MRI, PSMD, preterm, neonate, NODDI, DTI

## Abstract

Preterm birth is closely associated with cognitive impairment and generalized dysconnectivity of neural networks inferred from water diffusion MRI (dMRI) metrics. Peak width of skeletonized mean diffusivity (PSMD) is a metric derived from histogram analysis of mean diffusivity across the white matter skeleton, and it is a useful biomarker of generalized dysconnectivity and cognition in adulthood. We calculated PSMD and five other histogram based metrics derived from diffusion tensor imaging (DTI) and neurite orientation and dispersion imaging (NODDI) in the newborn, and evaluated their accuracy as biomarkers of microstructural brain white matter alterations associated with preterm birth. One hundred and thirty five neonates (76 preterm, 59 term) underwent 3T MRI at term equivalent age. There were group differences in peak width of skeletonized mean, axial, and radial diffusivities (PSMD, PSAD, PSRD), orientation dispersion index (PSODI) and neurite dispersion index (PSNDI), all *p* < 10^−4^. PSFA did not differ between groups. PSNDI was the best classifier of gestational age at birth with an accuracy of 81±10%, followed by PSMD, which had 77±9% accuracy. Models built on both NODDI metrics, and on all dMRI metrics combined, did not outperform the model based on PSNDI alone. We conclude that histogram based analyses of DTI and NODDI parameters are promising new image markers for investigating diffuse changes in brain connectivity in early life.

## 1. Introduction

Preterm birth is closely associated with a phenotype that includes cognitive impairment in childhood and cerebral white matter disease. White matter disease is apparent as diffuse changes in signal intensity on conventional MRI (1, 2), and alterations in diffusion MRI parameters based on the diffusion tensor [fractional anisotropy (FA), and mean, axial, and radial diffusivities, (MD, AD, RD)], and more recently, metrics based on biophysical models, such as neurite orientation and dispersion imaging (NODDI) (3, 4). These metrics have proven useful for making inferences about microstructural alteration of white matter that characterizes dysmaturity associated with preterm birth, for investigating upstream pathways to typical / atypical brain development, and for studying the anatomical bases of subsequent cognitive function in early life (5–13). However, deriving whole brain estimations of these parameters is often computationally expensive, there are uncertainties about which metric or combination of metrics best captures generalized white matter disease associated with preterm birth, and which is likely to be most useful for prognosis.

Peak width of skeletonized mean diffusivity (PSMD) is associated with processing speed—a foundational competence of cognition—in patients with small cerebral vessel disease, patients with Alzheimer's disease, healthy adults, and with a broader set of cognitive impairment (14, 15). In addition, it is a marker of widespread white matter tissue damage in multiple sclerosis (16). It works by calculating the width of the histogram of mean diffusivity of the skeletonized white matter (WM) tracts, thereby largely eliminating cerebrospinal fluid (CSF) contamination and focusing on the core of the main WM tracts. The framework is readily extensible to other DTI metrics (FA, RD, and AD) and to NODDI metrics.

Peak width of skeletonized DTI and NODDI metrics have potential to be useful biomarkers of preterm brain dysmaturation because several are known to be altered throughout white matter in association with preterm birth. Specifically, low FA and increased MD occur throughout the white matter in preterm infants at term equivalent age compared with term-born infants (4, 17–20), and NDI at term equivalent age it is negatively correlated with gestational age at birth (8). Additional advantages are that this framework is fully automated, only requires a single diffusion MRI acquisition, is computationally inexpensive, and has high inter-scanner reproducibility so could be used in clinical settings and for multi-center clinical trials (14).

In this work, we first optimized the PSMD pipeline described by Baykara et al. (14) for application to neonatal data in order to calculate values for PS- MD, FA, RD, AD, NDI, and ODI in early life. Next, based on the generalized dysconnectivity phenotype associated with preterm birth, we tested the hypothesis that infants born preterm would have differences in one or more of the histogram based metrics compared with infants born at term. Finally, we investigated the utility of these metrics by studying their relationship with gestational age at birth and testing their predictive ability in a classification task to discriminate between preterm and term brain images.

## 2. Methods

### 2.1. Participants and Data Acquisition

Participants were recruited as part of a longitudinal study designed to investigate the effects of preterm birth on brain structure and long term outcome (www.tebc.ed.ac.uk) (21). One hundred and thirty-five neonates underwent MRI at term equivalent age at the Edinburgh Imaging Facility: Royal Infirmary of Edinburgh, University of Edinburgh, UK.

A Siemens MAGNETOM Prisma 3 T MRI clinical scanner (Siemens Healthcare Erlangen, Germany) and 16-channel phased-array pediatric head coil were used to acquire: 3D T2-weighted SPACE (T2w) (voxel size = 1 mm isotropic) with TE 409 ms and TR 3,200 ms; and axial dMRI. dMRI was acquired in two separate acquisitions: the first acquisition consisted of 8 baseline volumes (b = 0 s/mm^2^ [b0]) and 64 volumes with b = 750 s/mm^2^, the second consisted of 8 b0, 3 volumes with b = 200 s/mm^2^, 6 volumes with b = 500 s/mm^2^ and 64 volumes with b = 2,500 s/mm^2^; an optimal angular coverage for the sampling scheme was applied (22). In addition, an acquisition of 3 b0 volumes with an inverse phase encoding direction was performed. All dMRI images were acquired using single-shot spin-echo echo planar imaging (EPI) with 2-fold simultaneous multislice and 2-fold in-plane parallel imaging acceleration and 2 mm isotropic voxels; all three diffusion acquisitions had the same parameters (TR/TE 3,400/78.0 ms). Images affected by motion artifact were re-acquired multiple times as required; dMRI acquisitions were repeated if signal loss was seen in 3 or more volumes.

Infants were fed and wrapped and allowed to sleep naturally in the scanner. Pulse oximetry, electrocardiography and temperature were monitored. Flexible earplugs and neonatal earmuffs (MiniMuffs, Natus) were used for acoustic protection. All scans were supervised by a doctor or nurse trained in neonatal resuscitation. Structural images were reported by an experienced pediatric radiologist (A.J.Q.) using the system described in Leuchter et al. (23), the exclusion criteria were the evidence of focal parenchymal injury (post-hemorrhagic ventricular dilatation, porencephalic cyst or cystic periventricular leukomalacia), or central nervous system malformation.

### 2.2. Data Pre-processing

All DICOM (Digital Imaging and Communication On Medicine) image files (dMRI and sMRI) were converted to the NIFTI (Neuroimaging Informatics Technology Initiative) format (24). Diffusion MRI processing was performed as follows: for each subject the two dMRI acquisitions were first concatenated and then denoised using a Marchenko-Pastur-PCA-based algorithm (25–27); eddy current, head movement and EPI geometric distortions were corrected using outlier replacement and slice-to-volume registration (28–32); bias field inhomogeneity correction was performed by calculating the bias field of the mean b0 volume and applying the correction to all the volumes (33). From the diffusion images we calculated the tensor (FA, MD, AD, and RD) and the NODDI (intracellular volume fraction [NDI] and the overall orientation dispersion index [ODI_TOT_]) maps (3, 34–36).

### 2.3. Atlas Construction

Images from 50 term born infants were used to create a multi-modality template (including T1w, T2w, FA, and tensor templates in addition to different parcellation schemes and tissue probability maps) using established methods (37). The final atlas is the Edinburgh Neonatal Atlas 50 (ENA50) (38). Prior to template creation, the structural images were processed using the minimal processing pipeline of the developing human connectome project (39, 40). To obtain the parcellation schemes, different neonatal atlases were registered to the T2w (38, 41–45). The mean b0 of each subject was co-registered to the T2w (46) and the inverse transformation was used to move all the maps to diffusion space (label maps, T1w and T2w).

The template was constructed using DTI-TK. In summary, it performs white alignment using a non-parametric, highly deformable, diffeomorphic registration method that incrementally estimates the displacement field using a tensor-based registration formulation (37). The resulting transformations were then applied to all the modalities. The final templates were obtained by averaging all the images of the same modality registered to the template space. For the parcellation maps, majority voting was used (47).

### 2.4. Peak Width of Skeletonized Water Diffusion MRI Derived Maps Calculation

All the subjects were registered to the tensor atlas using DTI-TK (48, 49). The tensor derived maps of each subject were calculated after registration and the NODDI metrics were propagated using the computed transformation. Then, the main skeleton of the FA template was created (50) by thresholding at 0.15, and individual FA maps were projected into this skeleton. Driven by the FA, the rest of the maps were projected onto the WM skeleton.

A custom mask was created by editing the skeleton mask to remove CSF and GM contaminated areas, and removing tracts passing through the cerebellum, the brainstem and the subcortical GM areas using ITK-Snap (51), using as a reference the custom mask created by Baykara et al. (14). The resulting skeletonized maps were then multiplied by the custom mask. Finally, the peak width of the histogram of values computed within the skeletonized maps was calculated as the difference between the 95th and 5th percentiles (14).

**Figure 1:**
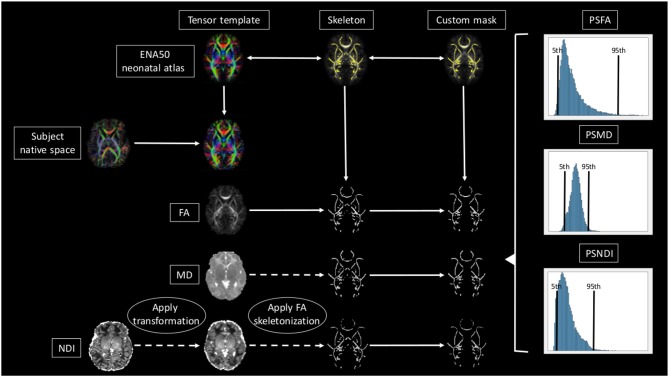
Overview of the full pipeline. For simplicity only NDI, MD, and FA are shown. The subject is registered to the ENA50 using a tensor registration. Then the DTI derived maps are generated and the transformation applied to the NODDI maps. Using FA as a conductor, the images are skeletonized, and finally, all images are multiplied by the custom mask.

A brief overview of the full pipeline can be seen in Figure 1.

### 2.5. Statistical Analysis

In the following analyses, metrics were adjusted for age a scan by fitting a liner model of each metric on GA at scan and retaining the residuals. We report Pearson's correlations between each of the residualized metrics and GA at birth in the whole sample. A D'Agostino and Pearson's test was used to assess the normality of the residualized imaging metrics. Group comparisons of residualized PS-MD, AD, RD, FA, NDI, and ODI were made using two-sample *t*-test for normally distributed variables and the Mann-Whitney U test for variables that did not have a normal distribution. Reported *p*-values were adjusted for the false discovery rate (FDR) using the Benjamini–Hochberg procedure. We then used the residualized metrics as predictors in a logistic regression model to discriminate between preterm and term born infants. We compared the performance of each metric individually and of three multivariate models including all the metrics, only DTI metrics and only NODDI metrics, respectively. We measured classification accuracy using a 30-repeated 10-fold cross validation, meaning that in each of 30 repetitions data are randomly split in 10-folds of which one in turn is used as a test set to assess the generalization ability of the model trained on the remaining 9-folds. Folds were stratified to preserve the proportion of term and preterm subjects of the whole sample. Accuracy was computed as the percentage of correctly classified subjects across folds and repetitions.

## 3. Results

Table 1 shows summary statistics of demographic characteristics of the study group.

**Table 1:** Demographic characteristics of the study group.

	**Preterm (*N* = 76)**	**Term (*N* = 59)**	**Term template (*N* = 50)**
Male:Female	43:33	31:28	25:25
Mean GA at birth/weeks (range)	29.48 (23.42–32)*	39.48 (36.42–42)	39.49 (37–42)
Mean GA at scan/weeks (range)	40.97 (38–44.56) *	41.84 (38.28–43.84)	41.89 (38.28–43.84)

Values marked with * are significantly different in preterm subjects with p < 0.01 after FDR correction.

**Figure 2:**
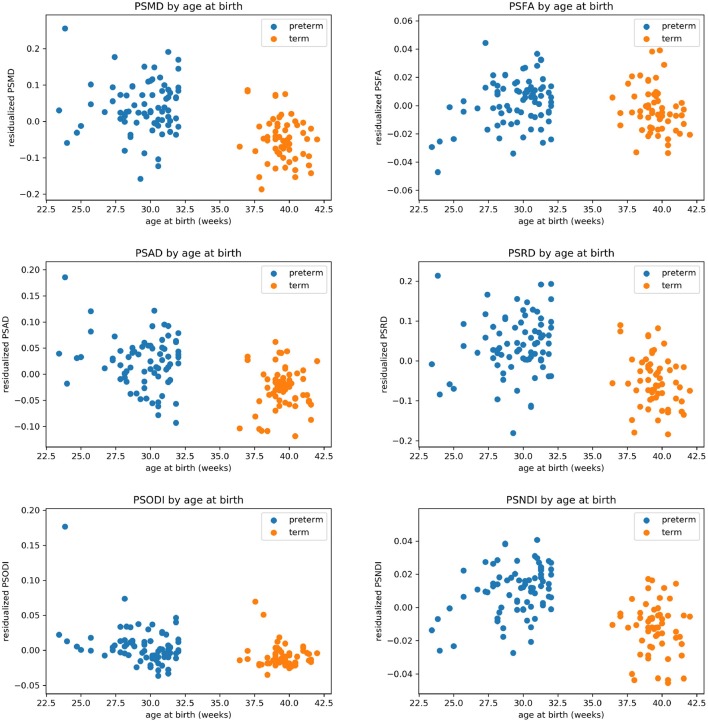
Scatter plots showing the relationship between each of the metric and gestational age at birth.

Table 2 shows the median, 25th and 75th percentiles, minimum and maximum values for each of the six histogram based metrics grouped by term and preterm categories, and Table 3 shows the mean 5th and 95th percentiles for the original metrics separated by group. Figure 2 shows the variation of each histogram based metric with respect to GA at birth and correlations between the metrics and GA at birth are reported in Table 4, together with results of group comparisons. With the exception of PSFA, all metrics were correlated with GA at birth (*p* < 0.01) and showed group differences in the term vs. preterm comparison (*p* < 0.01).

**Table 2:** Summary statistics for all metrics.

	**PSMD**	**PSFA**	**PSAD**	**PSRD**	**PSNDI**	**PSODI**
**TERM**						
Median	0.50	0.32	0.70	0.62	0.22	0.26
25%	0.48	0.31	0.68	0.57	0.21	0.25
75%	0.54	0.33	0.72	0.66	0.23	0.27
Min	0.38	0.29	0.61	0.48	0.18	0.23
Max	0.66	0.37	0.80	0.77	0.25	0.35
**PRETERM**						
Median	0.60	0.32	0.75	0.72	0.24	0.27
25%	0.56	0.32	0.72	0.67	0.23	0.27
75%	0.65	0.34	0.78	0.76	0.25	0.28
Min	0.45	0.28	0.63	0.54	0.19	0.24
Max	0.82	0.37	0.91	0.89	0.28	0.45

**Table 3:** Mean 5th and 95th percentiles of imaging metrics in preterm and term groups.

	**MD**	**FA**	**AD**	**RD**	**NDI**	**ODI**
**TERM**						
5%	1.04	0.15	1.37	0.77	0.07	0.02
95%	1.55	0.48	2.07	1.38	0.29	0.28
**PRETERM**						
5%	1.05	0.13*	1.37	0.79	0.05*	0.02
95%	1.65*	0.46*	2.12*	1.50*	0.29	0.29*

Values marked with * are significantly different in preterm subjects with p < 0.01 after FDR correction.

**Table 4:** Results for the correlation with GA and the classification task.

**Metric**	**Correlation with GA at birth**	**Group comparison**	**Classification accuracy**
PSMD	*r* = −0.52, *p* = 2.72 × 10^−10^	*t* = 7.59, *p* = 2.80 × 10^−11^	0.77 ± 0.09
PSFA	*r* = −0.11, *p* = 0.233	*t* = 2.02, *p* = 0.052	0.60 ± 0.05
PSAD	*r* = −0.49, *p* = 4.95 × 10^−9^	*t* = 6.40, *p* = 5.60 × 10^−9^	0.73 ± 0.11
PSRD	*r* = −0.50, *p* = 2.257 × 10^−09^	*t* = 7.45, *p* = 4.78 × 10^−11^	0.75 ± 0.09
PSNDI	*r* = −0.51, *p* = 8.50 × 10^−10^	*t* = 8.55, *p* = 4.56 × 10^−13^	0.81 ± 0.10
PSODI	*r* = −0.37, *p* = 1.39 × 10^−05^	*u* = 3272, *p* = 7.86 × 10^−06^	0.67 ± 0.17

Table 4 reports the cross-validation accuracy of each metric in the classification task (term vs. preterm). Four out of six metrics achieved at least 70% accuracy, with the exception of PSFA (60±5%) and PSODI (67±17%). PSMD and PSNDI obtained the best results among the DTI and NODDI metrics, respectively. Combining the metrics in a multivariate model increased only slightly the prediction accuracy: using all DTI metrics: 79±9% accuracy; using all NODDI metrics: 81±7% accuracy; using all metrics: 79±7% accuracy.

## 4. Discussion

We developed a pipeline for calculating peak width of skeletonized diffusion MRI derived metrics of the developing brain, and we show that five of these histogram based markers detect generalized white matter microstructural alteration associated with preterm birth. Calculation of these image markers is fully automated and computationally inexpensive, so peak width of skeletonized water diffusion metrics could have high value for research designed to investigate generalized dysconnectivity phenotypes in early life.

The NODDI and tensor derived metrics have been applied to different populations including healthy and aging adults (52, 53), patients with amyotrophic lateral sclerosis and Alzheimer's disease patients (54, 55), and to preclinical models (56, 57). In the neonatal MRI field, tensor derived metrics have been widely used to study the effects of prematurity on the brain (5), and several other factors, such as chronic lung disease, nutrition, prenatal drug exposure, among others (11, 13, 19, 58). In recent years NODDI metrics have been applied to neonatal data because of the added inference they offer with respect to microstructural organization and characteristics (59). They have revealed new insights into cortical maturation in perinatal life, and identified dysmaturation in newborns with congenital heart disease (59, 60). Recently, the tensor derived and NODDI metrics have been used together in integrated approaches, such as morphometric similarity networks (12, 61), but to our knowledge, this is the first time that DTI and NODDI metrics have been used within the peak width skeletonized framework for studying the developing brain.

We found that all the PS metrics with exception of the PSFA were higher for preterm infants at term equivalent age compared with values calculated from infants born at full term, meaning that the range of values was wider for the preterm population. However, the behavior of the metrics was different, which enables an inference about underlying tissue microstructure. For example MD has the same 5th percentile in term and preterm infants at term equivalent age but the 95th percentile is much higher in preterm group, whereas for NDI the opposite is true: both groups have the same 95th percentile, but the 5th percentile is much lower in preterm infants. Taken together, the metrics indicate higher variability in water content (toward higher values) and in intra-axonal volume (toward lower values) in preterm infant. This is consistent with lower myelination in preterm infants and/or less coherent WM organization (62), which is suggested by an overall higher PSODI for the preterm population. For RD the values in 95th and 5th percentile are higher in preterm than term, but the difference is much more accentuated in the 95th percentile, in agreement with PSMD. Increased PSAD in preterm infants at term equivalent age is consistent with altered axonal integrity, which is a feature of white matter disease in preterm infants. PSFA was the only metric that did not show a significant difference between groups, although there was a histogram shift (Table 3) such that term infants do have higher mean FA across the skeleton, which is a consistent finding across studies (4).

All the metrics, with exception of the PSFA and PSODI, achieved high accuracy (≥70%) in the classification task of preterm vs. term brain images. PSMD and PSNDI performed with greatest accuracy (Table 3), and this was not enhanced by combining multiple features in the same model. Different methods for preterm vs. term classification have been proposed with varying accuracy: 80% (63) or 92% (12). However, previous methods usually require long acquisitions and/or complicated processing frameworks. The main advantage of the histogram based framework is that it is possible to calculate measures from standard diffusion MRI acquisition and with relatively simple processing, making it suitable for large scale multi-site studies (14).

Application of histogram based methods to neonatal data required some modifications to the original framework proposed by Baykara et al. First, we optimized the method to operate in a specific neonatal space, as opposed to the MNI152 co-ordinate system (64). For doing this, a neonatal template was created (ENA50) and used as a common space for the whole process. The registration method was also changed: the original method uses FNIRT (65) because it is based in the main TBSS framework (50). Due the nature of the tensor-based neonatal atlas, we are able to use a tensor-based registration (48, 49) with a three-step registration, adding a rigid step at the beginning (19). This method has been shown to improve the alignment of WM tracts in neonatal data (10, 19, 66). One of the main advantages of the proposed framework, is that due to the multi-modal nature of the ENA50 (FA, T1-weighted, T2-weighted and tensor templates) the pipeline can be easily modified to change the registration process for any of the available intensity-based algorithms (41, 65, 67–69).

Histogram based analyses of DTI and NODDI metrics offer tractable markers that could be used to investigate generalized white matter connectivity in other neonatal populations at risk of atypical brain development and the extensible nature of the framework means that it could be applied to other myelin sensitive metrics not derived from diffusion, such as T1w/T2w (70) or g-ratio (71). Future work could investigate the utility of histogram based metrics for assessing the impact of perinatal exposures and co-morbidities on brain tissue development, and their predictive value for cognitive and behavioral outcomes in children at risk of impairment. Furthermore, their possible utility in clinical settings, providing summary information about WM microstructure from MRI datasets acquired on different scanners, and as potential biomarkers in neuroprotection trials should be evaluated.

## 5. Conclusion

In this work, we introduce an age-specific pipeline for calculation of peak width of skeletonized MD, RD, AD, FA, NDI, and ODI of the neonatal brain. We found that these histogram based metrics, which represent generalized water content, myelination, and complexity of dendrites and axons across the WM skeleton, are altered in association with preterm birth. PSMD and PSNDI appear to be the most promising biomarkers due to their relative ease of computation compared with other methods, and their comparable accuracy.

## Data Availability Statement

The atlas with templates can be found at https://git.ecdf.ed.ac.uk/jbrl/ena and the code necessary to calculate histogram based metrics is at https://git.ecdf.ed.ac.uk/jbrl/psmd. Reasonable requests for original image data will be considered through the BRAINS governance process: www.brainsimagebank.ac.uk (72).

## Ethics Statement

The studies involving human participants were reviewed and approved by UK National Research Ethics Service. Written informed consent to participate in this study was provided by the participants' legal guardian/next of kin.

## Author Contributions

MB and PG designed the experiments and wrote the first draft of the manuscript. MB processed the data. PG did the statistical analyses. GS, DS, GL, AQ, and MT acquired the data. EW contributed to the analysis of data. JB and MEB provided help with the interpretation and wrote the manuscript. All authors revised and commented on the manuscript.

### Conflict of Interest

The authors declare that the research was conducted in the absence of any commercial or financial relationships that could be construed as a potential conflict of interest.
